# Domestic Correspondence

**Published:** 1890-09

**Authors:** 


### Domestic Correspondence.

TO THE EDITOR:

THE Colorado State Dental Association held its Fourth Annual Meeting in Denver, June 24 to 26. The following papers were read and discussed: "Dental Caries as influenced by Acids," Dr. H. A. Fynn, Central; "Dentistry of To-Day," Dr. M. H. Smith, Colorado Springs; "Microbes," Dr. A. H. Sawins, Denver; "Separating Teeth Previous to Filling,—Is it Advisable?" Dr. W. E. Griswold, Denver; "Does Attitude affect the Teeth?" Dr. C. F. Dodge, Leadville; "Fees," Dr. J. M. Porter, Denver; "Pulpless Teeth," Dr. H. P. Kelley, Denver; "Pyorrhœa," Dr. P. T. Smith, Denver.

Our next session will be held the first Tuesday in next June, and any brother practioner who may be making a tour of the Rockies at that time in quest of health or for any other purpose will be cordially welcomed. Please remember that the number of dentists throughout the whole country who take an active, progressive part in Dental Associations is comparatively few, and when you are coming as far west as Missouri and Kansas do not forget to come on to Colorado, the Switzerland of America.

J. H. PARSONS, D.D.S.,
*Corresponding Secretary.*

BOULDER, COLORADO.

To the Editor:

The Twenty-sixth Annual Meeting of the Missouri State Dental Association was held at Pertle Springs, Warrensburg, Mo., July 8, 9, 10, 11, 1890.

President Dr. Henry Fisher called the meeting to order.

The committee on resolutions to the memory of Dr. A. Noland presented the following, which were adopted by the Association:

To the Members of the Missouri State Dental Association:

Whereas, Death has removed from our ranks our beloved brother and co-laborer, Dr. A. Noland, of Monroe City, on the 22d of January, 1890; and

Whereas, Dr. Noland was a faithful student, an honored laborer, and worked hard to raise the standard of our profession in this State; therefore,

*Resolved*, That this Association mourn with sorrow the loss sustained.

*Resolved*, That this Association hereby tender his bereaved family its heartfelt sympathies and condolence in this their sad bereavement, and may that God in whom he so implicitly trusted speak peace to their sad hearts in their distress.

*Resolved*, That a copy of these resolutions be sent to the family of our deceased brother and to the dental journals for publication.

B. Q. Stevens,
G. M. Risley,
Jas. L. Leavel,
*Committee.*

The committee to draft resolutions on the death of Dr. Judd presented the following, which were adopted:

Whereas, The recent death of Dr. Homer Judd brings to mind his activity and influence in the organization of this Association, and his subsequent labor in extending its usefulness, to the great benefit of the profession in this State; and,

Whereas, His noble character, energy, and his literary attainments entitle him to an exalted place on the scroll of deceased members; therefore,

*Resolved*, That in the death of Dr. Judd this Association has lost an honored member, whose professional character and example we emulate, and whose memory we ever hold dear.

*Resolved*, That the heartfelt sympathy and condolence of this Association be tendered the family of our departed brother in their sad bereavement.

*Resolved*, That a copy of these resolutions be sent to the family and to the dental journals for publication.

W. H. Eames,
C. W. Spalding,
J. C. Goodrich,
Pertle Springs, July 9, 1890. *Committee.*

Drs. Price, T. W. Reed, and F. Swap were appointed a committee to draft suitable farewell resolutions to Dr. Spalding, and reported as follows:

MR. PRESIDENT AND MEMBERS OF THE MISSOURI STATE DENTAL ASSOCIATION:

GENTLEMEN,—We, your committee appointed to draft resolutions expressive of the pleasure of this Association afforded by the presence of Dr. C. W. Spalding, and of regret that he will soon sever his social connections with us, most respectfully submit the following resolutions:

*Resolved,* That with a full appreciation of Dr. Spalding's virtues as a gentleman, his high moral character as a man, his eminent qualifications and invaluable counsel as a professional brother, we tender to him our most sincere thanks for his presence at this meeting (probably the last time we shall all meet him on this earth); we deeply regret that he should feel it necessary to sever his social connection with us.

*Resolved,* That we fully realize the fact that no one has done more than he to advance the standard of our profession and the best interests of this Association, which will ever, by us, be remembered and cherished with grateful hearts.

*Resolved,* That in his departure he takes with him our most earnest wishes for his success, prosperity, and happiness; and may kind Providence ever watch over, guide, and shield him.

*Resolved,* That these resolutions be spread upon the records of this Association, a copy, properly engrossed, be presented to Dr. Spalding, and one sent to each of the dental journals for publication.

JAS. A. PRICE,

T. W. REED,

F. SWAP,

*Committee.*

The election of officers resulted as follows: President, Dr. J. F. McWilliams, Mexico; Vice-President, Dr. Geo. L. Shephard, Sedalia; Second Vice-President, Dr. W. H. Buckley, Liberty; Recording Secretary, Dr. John G. Harper; Corresponding Secretary, Dr. William Conrad; Treasurer, Dr. James A. Price, Weston.

*Board of Censors.*—Drs. J. G. Hollingworth, W. L. Reed, Chas. L. Hungerford.

*Committee on Ethics.*—Drs. N. H. Gaines, C. V. Huff, J. W. Aikin.

*Publication Committee.*—Drs. E. E. Shattuck, H. S. Lowry, W. E. Tucker, Law; Jas. A. Price, Weston.

*Committee on New Appliances.*—Dr. J. M. Austin, St. Joseph.

*Executive Committee.*—Dr. William Conrad, Dr. Henry Fisher, and Dr. J. W. Whipple, St. Louis.

*Supervisor of Clinics.*—Dr. A. J. Prosser, St. Louis.

Next place of meeting, Louisiana, Mo., first Tuesday after July 4, 1891.

WM. CONRAD,
*Corresponding Secretary.*

321 N. GRAND AVENUE.

